# Examining the profile of high-potency cannabis and its association with severity of cannabis dependence

**DOI:** 10.1017/S0033291715001178

**Published:** 2015-07-27

**Authors:** T. P. Freeman, A. R. Winstock

**Affiliations:** 1 Clinical Psychopharmacology Unit, University College London, London, UK; 2 Institute of Psychiatry, King's College London, Camberwell, UK

**Keywords:** Addiction, cannabidiol, cannabis, delta-9-tetrahydrocannabinol, dependence, marijuana

## Abstract

**Background:**

Cannabis use is decreasing in England and Wales, while demand for cannabis treatment in addiction services continues to rise. This could be partly due to an increased availability of high-potency cannabis.

**Method:**

Adults residing in the UK were questioned about their drug use, including three types of cannabis (high potency: skunk; low potency: other grass, resin). Cannabis types were profiled and examined for possible associations between frequency of use and (i) cannabis dependence, (ii) cannabis-related concerns.

**Results:**

Frequent use of high-potency cannabis predicted a greater severity of dependence [days of skunk use per month: *b* = 0.254, 95% confidence interval (CI) 0.161–0.357, *p* < 0.001] and this effect became stronger as age decreased (*b* = −0.006, 95% CI −0.010 to −0.002, *p* = 0.004). By contrast, use of low-potency cannabis was not associated with dependence (days of other grass use per month: *b* = 0.020, 95% CI −0.029 to 0.070, *p* = 0.436; days of resin use per month: *b* = 0.025, 95% CI −0.019 to 0.067, *p* = 0.245). Frequency of cannabis use (all types) did not predict severity of cannabis-related concerns. High-potency cannabis was clearly distinct from low-potency varieties by its marked effects on memory and paranoia. It also produced the best high, was preferred, and most available.

**Conclusions:**

High-potency cannabis use is associated with an increased severity of dependence, especially in young people. Its profile is strongly defined by negative effects (memory, paranoia), but also positive characteristics (best high, preferred type), which may be important when considering clinical or public health interventions focusing on cannabis potency.

## Introduction

There is huge variation in the types of cannabis (marijuana) available worldwide (UNODC, 2014). This is evident in illicit markets and also legal ones. For example, an unprecedented number of cannabis products and preparations are now available in Colorado (Coombes, 2014). By contrast, sales in Uruguay may be restricted to five strains only, with an upper limit on potency (Coombes, 2014).

Cannabis potency is typically judged according to concentrations of delta-9-tetrahydrocannabinol (THC), the primary psychoactive constituent in cannabis. However, the cannabis plant contains many other cannabinoids, most notably cannabidiol (CBD). These other cannabinoids (and possibly other plant chemicals known as terpenoids; Russo, 2011) contribute to potency by moderating the effects of THC. For example, CBD can block or dampen the effects of THC across a range of domains (Zuardi *et al.* 1982; Morgan & Curran, 2008; Morgan *et al.* 2010*a*,*b*, 2012; Englund *et al.* 2012; Hindocha *et al.* 2015). These findings concur with users’ ratings of cannabis potency, which are positively correlated with THC and negatively with CBD (Freeman *et al.* 2014).

Natural cannabinoid synthesis (and therefore cannabis potency) is influenced by a range of factors including genetics, growing conditions (especially light), harvest time, the part of the plant used, drying, storing and processing (Potter, 2014). Most products can be classified into three broad types: (1) high potency – indoor-grown floral material of unfertilized plants, whereby energy is diverted from seed production to cannabinoid synthesis (‘skunk’, ‘sinsemilla’; meaning ‘without seeds’); (2) low potency – outdoor-grown imported floral material (‘herbal’, ‘grass’, ‘weed’); and (3) compressed blocks of plant matter (‘resin’, ‘hashish’). Skunk is characterized by the highest THC content (~15%), followed by imported herbal/grass (~9%) and then resin (~5%), although there is considerable variation within these categories (Hardwick & King, 2008). Concentrations of CBD are typically low or completely absent in skunk and other herbal/grass preparations. By contrast, resin/hashish (and presumably landrace populations of cannabis plants) typically contain comparable quantities of THC and CBD (Potter *et al.* 2008). Thus, indoor-grown floral cannabis (skunk) is the clearly most potent type of cannabis (followed by imported herbal/grass, and then resin/hashish), and might be expected to be most strongly associated with any adverse effects of cannabis use. This is currently an under-researched area, although preliminary evidence suggests that regular use of high-potency (skunk) cannabis is predictive of first-episode psychosis (Di Forti *et al.* 2009) and an earlier onset of psychosis, particularly among daily users (Di Forti *et al.* 2013). By contrast, resin/hashish is not linked to an increased risk of psychosis, even among daily users (Di Forti *et al.* 2015).

It is estimated that 3.8% of the world's population used cannabis in the last year (UNODC, 2014) and this figure has remained relatively stable in the last decade. In England and Wales, however, prevalence of last year use dropped from 10.7% to 6.6% between 2002/2003 and 2013/2014 (Home Office, 2014). Despite this overall reduction in use, demand for cannabis in addiction-treatment services has continued to rise across the same time period: between 2005/2006 and 2013/2014 new admissions for cannabis rose from 7579 to 11 821 in adults (NDTMS, 2014) and from 9043 to 13 659 among under 18s (NDTMS, 2015). There are now more first-time clients for cannabis treatment in Europe than any other illicit drug (EMCDDA, 2014).

One possible explanation for these trends is an increase in cannabis potency. Data from cannabis seizures have documented rising THC concentrations in the UK. This is predominantly due to an increase in the availability of high-potency, indoor-grown (skunk) cannabis which made up 15% of police seizures in 1999–2002 (King *et al.* 2004), 55% in 2004–2005 (Potter *et al.* 2008) and 81% in 2007–2008 (Hardwick & King, 2008). These trends are matched by seizure data across Europe (EMCDDA, 2014). Cannabis users titrate (use less) as THC rises but only partially (Freeman *et al.* 2014; van der Pol *et al.* 2014) and not in response to CBD (Freeman *et al.* 2014). It is therefore possible that repeated exposure to high THC concentrations, and little if any CBD, may have increased users’ dependence on cannabis.

In this study, we recorded detailed information on use of and experiences with different types of cannabis through an online drug survey. This approach made it possible to recruit a large sample who had used all of three different cannabis types (skunk, other grass, resin) within the last 12 months, permitting within-subject comparison of cannabis types. We aimed to test the hypothesis that severity of dependence, and concerns about cannabis use, are more strongly associated with use of high-potency than low-potency cannabis. Additionally, we explored users’ experiences of each type of cannabis in relation to effects on memory, paranoia, quality of high, preference, value for money and availability.

## Method

### Design and participants

An online cross-sectional drugs survey, The Global Drug Survey, was conducted in November 2009 as reported elsewhere (Winstock *et al.* 2011). All participants confirmed that they were aged ⩾18 years, and consented for the information they gave to be analysed. Ethical approval was received from the joint South London and Maudsley and Institute of Psychiatry National Health Service (NHS) Research Ethics Committee.

### Assessments

The survey collected demographic data and detailed information on use of and experience with a number of substances. The data presented and analysed in this report is the UK data only; cases living in England, Scotland, Wales, and Northern Ireland form the UK dataset. Rather than recording information on cannabis use generally (e.g. age when cannabis was first used, days of cannabis use per month), separate questions were provided for (1) resin, (2) skunk grass (hereafter ‘skunk’), (3) grass other (hereafter ‘other grass’). This enabled each type to be rated as a separate drug. The following information was collected for each type of cannabis:

### Comparing use of cannabis types

Used in the last 12 months? (yes/no).

Days used in the last month.

How long does ⅛th last you (in days).†

How many joints from ⅛th?

### Profiling cannabis types

Respondents who had used all three cannabis types in the last 12 months were asked to choose one type for each of the following questions:

Which gives the best high?

Which is the best value for money?

Which is most likely to get you paranoid?

Which is most likely to affect your memory?

Which is your preferred type?

Which is most available?

### Route of administration

Respondents were asked whether they had ever used cannabis using the following methods: smoked in joint without tobacco, smoked in joint with tobacco, smoked in bong/water pipe without tobacco, smoked in bong/water pipe with tobacco, eaten/cooked, used in a vaporizer.

### Severity of dependence and cannabis-related concerns

These questions were assessed with reference to cannabis use generally. Cannabis dependence was assessed using the Severity of Dependence Scale (SDS; Gossop *et al.* 1995), which was adapted for the survey with abbreviated response options as shown below. Scores can range from 0 to 15, and scores ⩾3 on the original scale indicate dependence on cannabis (Swift *et al.* 1998).

(1) Do you ever think your use of cannabis is out of control? [never (0); sometimes (1); often (2); always (3)].
(2) Does the prospect of missing a smoke make you very anxious or worried? [never (0); sometimes (1); often (2); always (3)].
(3) Do you worry about your use of cannabis? [never (0); sometimes (1); often (2); always (3)].
(4) Do you wish you could stop? [never (0); sometimes (1); often (2); always (3)].
(5) How difficult would you find it to stop or go without? [not difficult (0); quite difficult (1); very difficult (2); impossible (3)].

Additionally, the following questions were asked (yes/no):

Have you ever discussed your cannabis use with a healthcare professional?

Have you ever thought you might need treatment for your cannabis use?

Have you ever sought treatment for cannabis use?

Have you ever tried to stop smoking cannabis?

Participants were also asked about a range of concerns relating to their cannabis use:

We are interested in what worries you about smoking cannabis. Please rate the following possible health-related consequences of smoking cannabis on a scale of 1–10, where 1 = no concern for you and 10 = big concern for you: cancer, chronic lung disease, effect on memory, effect on mental health, legal issues, effect on relationships, effect on work or study, lack of motivation.

### Statistical analysis

Repeated-measures ANOVA models were used to compare each cannabis type for indices of use. *Post-hoc t* tests were corrected locally using the Bonferroni method. χ^2^ tests were used for comparing the profile of cannabis types. Current age was split into quartiles (<21, 21–22, 23–27 and >27 years) for analysis of first use, and profile of effects. Pearson correlational analyses were used to establish associations between cannabis use variables and SDS scores. Multiple regression was used to predict severity of cannabis dependence and cannabis-related concerns from indices of cannabis use. Analysis of gender was coded as female = 1, male = 2, and age was entered as a continuous variable. For all regression models, bias-corrected accelerated 95% confidence intervals (CIs) were estimated using 10 000 boostrapping samples.

## Results

### Demographics

Data were available for 2514 respondents. In the last year, prevalence of use was 72.5% for skunk, 68.6% for other grass, and 58.7% for resin cannabis preparations. Thirty-seven per cent (929 respondents) had used all three cannabis preparations in the last year; all further analyses were conducted in this sample. These participants had a mean age of 24.25 (s.d.=6.86) years and 70.2% were male. Routes of administration (ever used/most common use) were as follows: smoked in joint with tobacco (98.9%/85.2%), smoked in joint without tobacco (75.5%/5.5%), smoked in bong/water pipe with tobacco (69.6%/3.3%), smoked in bong/water pipe without tobacco (85.2%/4.5%), eaten/cooked (80.1%/1.1%), used in a vaporizer (36.8%/0.4%).

### Comparing use of cannabis types (Table 1)

Participants reported differences in the number of days they had used each type of cannabis in the last month (*F*_2,761_ = 38.332, *p* < 0.001, *ŋ*_p_^2^ = 0.087). Skunk was used for more days than other grass (*p* < 0.001) and resin (*p* < 0.001), while other grass was used for more days than resin (*p* < 0.001). No differences were found for the number of days to smoke ⅛th (*F*_2,895_ = 1.655, *p* = 0.197, *ŋ*_p_^2^ = 0.003). Differences emerged for the number of joints made out of ⅛th (*F*_2,893_ = 62.710, *p* < 0.001, *ŋ*_p_^2^ = 0.108), reflecting a larger number of joints made from resin compared to skunk (*p* < 0.001) or other grass (*p* < 0.001); a similar number of joints were made for skunk and other grass (*p* = 0.078). There were also differences in age of first use (*F*_2,1432_ = 41.059, *p* < 0.001, *ŋ*_*p*_^*2*^ = 0.043); resin was used earlier than skunk (*p* < 0.001) and other grass (*p* = 0.002); while other grass was used earlier than skunk (*p* < 0.001).

**Table 1.** Comparing use of three cannanbis types

	Skunk	Other grass	Resin
Days used in the last month^a^	14.05 (10.68)	11.46 (10.43)	9.45 (9.90)
Days taken to smoke 3.5 g	8.55 (29.28)	7.31 (24.49)	8.14 (19.80)
Number of joints made out of 3.5 g^a^	7.93 (5.12)	7.63 (4.87)	9.41 (5.70)
Age first used^a^	15.90 (3.70)	15.38 (2.55)	15.18 (2.67)

Values given are mean (s.d.) scores.

a Difference across cannabis types at *p* < 0.001.

**Fig. 1.**
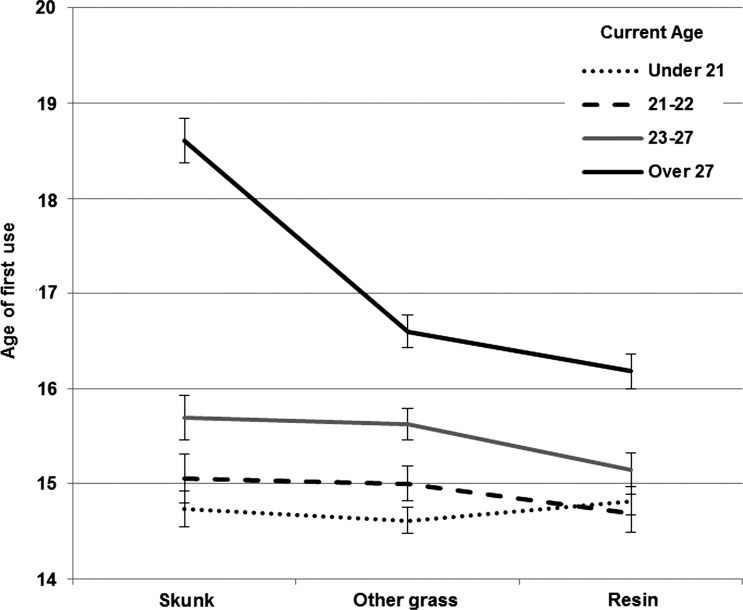
Current age and first use of cannabis. Young people in the sample (currently under 23) were exposed to all three types of cannabis at similar ages. Older people were exposed to resin earlier than other types of cannabis, and skunk use was markedly delayed in the over 27's. These results support a shift in the relative availability of resin and skunk over time.

The later age of skunk onset might be attributable to its low availability at the time when this sample first tried cannabis (e.g. 15% prevalence in 1999–2002; King *et al.* 2004). We explored this possibility by adding a between-subject factor of current age, split into quartiles, into the model (see Fig. 1). This revealed a cannabis type × age interaction (*F*_5,1468_ = 29.456, *p* < 0.001, *ŋ*_p_^2^ = 0.089) as well as effects of cannabis type (*F*_2,1468_ = 53.598, *p* < 0.001, *ŋ*_p_^2^ = 0.056) and age (*F*_3,900_ = 42.331, *p* < 0.001, *ŋ*_p_^2^ = 0.124). *Post-hoc* tests showed that in young people (under 21's and 21- to 22-year-olds), all three types of cannabis were first tried at similar ages (all *p*'s > 0.06). By contrast, 23- to 27-year-olds had tried resin earlier than other grass (*p* < 0.001) and skunk (*p* = 0.010), which were both tried at an equivalent age (*p* = 1.000). In the over 27's, there was a marked delay in first trying skunk relative to other grass (2.00 years, *p* < 0.001) and resin (2.42 years, *p* < 0.001), and resin was again tried earliest (*p* = 0.002 compared to other grass). These data are consistent with a shift in the relative availability of resin and skunk over time, alongside a tendency for younger people to try cannabis at an earlier age.

### Profiling cannabis types (Fig. 2)

Ratings differed across the three cannabis types for ‘best high’ (χ^2^_2_ = 539.919, *p* < 0.001), ‘value for money’ (χ^2^_2_ = 126.788, *p* < 0.001), ‘most likely to get you paranoid’ (χ^2^_2_ = 719.880, *p* < 0.001), ‘most likely to affect your memory’ (χ^2^_2_ = 838.049, *p* < 0.001), ‘preferred type’ (χ^2^_2_ = 246.739, *p* < 0.001), and ‘most available’ (χ^2^_2_ = 360.622, *p* < 0.001). As shown in Fig. 1, skunk scored the highest for ‘best high’, ‘most likely to get you paranoid’, ‘most likely to affect your memory’, ‘preferred type’, ‘most available’. Among these, resin scored above other grass for ‘best high’ (χ^2^_1_ = 7.879, *p* = 0.005), ‘most likely to get you paranoid’ (χ^2^_1_ = 18.447, *p* < 0.001) and ‘most likely to affect your memory’ (χ^2^_1_ = 44.445, *p* < 0.001). By contrast, resin scored lower than other grass for ‘most available’ (χ^2^_1_ = 30.201, *p* < 0.001) and they both scored equally for ‘preferred type’ (χ^2^_1_ = 1.011, *p* = 0.315). In terms of ‘value for money’, resin was rated the highest, above skunk (χ^2^_1_ = 15.334, *p* < 0.001), which in turn scored higher than other grass (χ^2^_1_ = 58.911, *p* < 0.001). The same pattern of results was found when the sample was split according to gender or age quartiles.

**Fig. 2.**
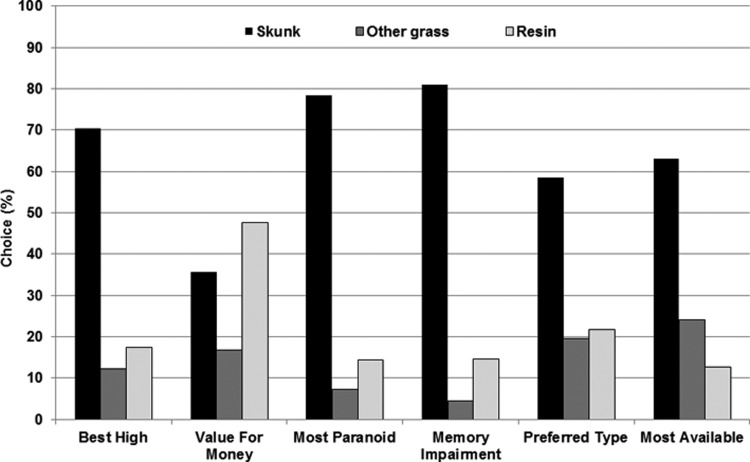
Characteristics of three cannabis types. Skunk was the predominant choice for all attributes apart from value for money.

### Severity of dependence and cannabis-related concerns

Exploratory correlations were conducted between SDS scores and all 12 indices of use (for skunk, other grass and resin: age of first use, days used in the last month, days taken to smoke ⅛th, number of joints from ⅛th). For days used in the last month, Pearson's *r* values reflected a medium-large effect size for skunk (*r* = 0.432) and small-medium effect sizes for other grass (*r* = 0.290) and resin (*r* = 0.247). For all other indices of use, effect sizes were small (all *r*'s ⩽0.155). Scores for severity of dependence (and individual concerns about cannabis) were therefore regressed onto days of skunk, other grass, and resin use in the last month.

Four hundred and three respondents had used each of the three cannabis types at least once in the last month; the following analyses were conducted in those individuals. Within their lifetime, 23.0% had discussed their cannabis use with a healthcare professional, 17.8% thought they might need treatment for their cannabis use, 5.3% had sought treatment for cannabis use, and 47.4% had tried to stop smoking cannabis. On the SDS, they had a mean score of 2.82 (s.d. = 3.29). Scores ranged from 0 to 14 (out of a maximum of 15) and quartiles were 0.00, 2.00, and 4.50. When classified using the cut-off of ⩾3 (Swift *et al.* 1998), 38% of the sample currently met criteria for cannabis dependence.

As shown in Table 2, frequency of cannabis use in the last month predicted severity of dependence, accounting for 14.4% of the variance in these scores. This was driven by skunk; no associations emerged for other grass or resin. We additionally investigated whether this effect was moderated by gender or age. Adding gender and age into the model (step 2) did not account for additional variance, but including them as moderators of skunk use (step 3) improved model fit. Removing redundant predictors (step 4) did not result in a loss of variance explained and accounted for a total of 15.5%. SDS scores increased (*b* = 0.254, 95% CI 0.161–0.357, *p* < 0.001) for each additional day of skunk per month. This relationship became stronger as age decreased (*b* = −0.006, 95% CI −0.010 to −0.002, *p* = 0.004). SDS scores also increased with age (*b* = 0.081, 95% CI 0.014–0.170, *p* = 0.039).

**Table 2.** Predicting severity of cannabis dependence from frequency of use

		95% CI		
	*b*	Lower	Upper	*p*
**Step 1**				
Total *R*^2^ = 0.144, *p* < 0.001				
Constant	1.046	0.636	1.473	
Skunk	**0.093**	**0.048**	**0.139**	**<0.001**
Other grass	0.020	−0.029	0.070	0.436
Resin	0.025	−0.019	0.067	0.245
***Step 2***				
*ΔR*^2^ = 0.003, *p* = 0.553				
Total *R*^2^ = 0.147, *p* < 0.001				
Constant	1.746	0.413	2.981	
Skunk	**0.096**	**0.051**	**0.143**	**<0.001**
Other grass	0.018	−0.030	0.069	0.477
Resin	0.025	−0.018	0.067	0.245
Age	−0.003	−0.046	0.050	0.905
Gender	−0.380	−1.064	0.317	0.268
***Step 3***				
*ΔR*^2^ = 0.023, *p* = 0.005				
Total *R*^2^ = 0.170, *p* < 0.001				
Constant	0.913	−0.874	2.537	
Skunk	**0.146**	**0.018**	**0.278**	**0.024**
Other grass	0.014	−0.034	0.062	0.584
Resin	0.022	−0.021	0.064	0.298
Age	**0.078**	**0.012**	**0.156**	**0.037**
Gender	−1.030	−2.142	0.094	0.058
Age × skunk	−**0.006**	−**0.010**	−**0.002**	**0.003**
Gender × skunk	0.052	−0.019	0.121	0.160
***Step 4***				
*ΔR*^2^* = −*0.015, *p* = 0.138				
Total *R*^2^ = 0.155, *p* < 0.001				
Constant	−0.733	−2.865	0.922	
Skunk	**0.254**	**0.161**	**0.357**	**<0.001**
Age	**0.081**	**0.014**	**0.170**	**0.039**
Age × skunk	−**0.006**	−**0.010**	−**0.002**	**0.004**

CI, Confidence interval.

Significant predictor variables are shown in bold.

Days of skunk use, but not other grass or resin, predicted higher Severity of Dependence Scale scores. The relationship between skunk use and severity of dependence became stronger as age decreased.

Mean (s.d.) scores for cannabis-related concerns about cannabis were as follows (presented in descending order of concerns). Memory: 4.98 (2.87); work or study: 4.58 (2.98); mental health: 4.42 (3.16); motivation: 3.97 (3.50); chronic lung disease: 3.85 (2.76); cancer: 3.70 (2.75); relationships: 3.33 (2.71); legal issues: 3.01 (2.66). Frequency of cannabis use (skunk, other grass, resin) did not predict scores for any of these concerns.

## Discussion

This study compared the profile of three cannabis types and their associations with cannabis dependence. Our findings clearly show that use of high-potency (skunk) but not low-potency (other grass, resin) cannabis is associated with an increased severity of dependence, especially in young people. Furthermore, the profile of high-potency (skunk) cannabis was marked in terms of negative effects (memory and paranoia) but also positive characteristics (preferred type and best high). It was also rated as the most available type, but was not the best value for money.

The past decade has seen a huge increase in the prevalence of high-potency (skunk-type) cannabis in England and Wales (King *et al.* 2004; Hardwick & King, 2008; Potter *et al.* 2008; Freeman *et al.* 2014) alongside rising demand for cannabis treatment in addiction services (NDTMS, 2014, 2015). Our findings are consistent with these observations, and are the first to our knowledge reporting a link between cannabis potency and severity of drug dependence. Thus, clinically, it might be useful (and desirable) to encourage skunk users at risk of/experiencing dependence to move to less potent forms of cannabis if they are not motivated to quit.

Younger people were especially vulnerable, displaying a stronger relationship between extent of skunk use and severity of dependence. This is in agreement with observations that more under 18's seek treatment for cannabis than all adults combined, unlike any other drug (NDTMS, 2014, 2015). Young people in our sample were also exposed to skunk from an earlier age; older adults tried resin first and had not tried skunk until an average of 2.42 years later. Given that these changes in the illicit market may have increased rates of cannabis dependence in the UK, it will be important to evaluate the impact of careful regulation of cannabis potency (e.g. as planned in Uruguay) and other legislative changes (e.g. in the US) on cannabis dependence.

One explanation for our findings is that greater THC exposure enhances the dependence-forming properties of cannabis. This interpretation is in keeping with preclinical research showing that THC is reinforcing in a dose-dependent manner (Tanda *et al.* 2000; Justinova *et al.* 2003). Titration by cannabis users appears to counteract higher THC concentrations, but only partially (Freeman *et al.* 2014; van der Pol *et al.* 2014). As a result, episodic use of high-potency cannabis will typically deliver larger doses of THC.

Interestingly, people in this study added less cannabis to their joints (based on the number of joints made out of 3.5 g) when using resin compared to other types. This may have further reduced their dose of THC, in addition to the low potency typical of resin. However, they took a similar number of days to smoke 3.5 g in total – perhaps suggesting that resin users smoke more joints with smaller amounts of cannabis (and possibly more tobacco), resulting in similar consumption of raw cannabis overall. The presence of CBD may also be relevant. Cannabis with a high ratio of CBD:THC (i.e. resin) reduced attentional bias to drug cues (a process implicated in addiction; Field & Cox, 2008) relative to low CBD:THC cannabis (i.e. skunk) (Morgan *et al.* 2010*b*). CBD was also found to reduce symptoms of cannabis withdrawal in an open-label case study (Crippa *et al.* 2012).

Although our results support a relationship between cannabis potency and severity of dependence, they do not imply a causal relationship, and many other factors are likely to be involved. For example, a prospective study found no independent associations between indices of cannabis use (including preferred type and THC concentrations) and subsequent incidence of dependence (van der Pol *et al.* 2013). This study differed from ours in a number of respects, and included a number of additional predictors (e.g. socio-demographic, vulnerability and stress factors). Additionally, it used a between-subjects comparison of preferred cannabis type and potency as opposed to our within-subject analysis.

Contrary to our expectations, degree of cannabis use did not predict level of concerns about cannabis (memory, mental health, work or study, relationships, motivation, chronic lung disease, cancer, legal issues). This might reflect the varying susceptibility to cannabis-related harms between individuals. Another possible explanation is that more frequent users hold the belief that their use is not problematic. These findings also suggest that all levels of use can be associated with modest health concerns. This may imply that infrequent users who are not currently using treatment services are nevertheless worried about the effects of cannabis, and might benefit from help at an individual or population-based level.

It is also noteworthy that memory emerged as the strongest concern about cannabis use in this study, as this was also the most defining feature of high-potency cannabis. This is consistent with the high THC, low CBD profile of skunk. When acutely administered, THC produces robust and dose-dependent impairments in verbal memory (Curran *et al.* 2002; D'Souza *et al.* 2004) and these impairments can be ameliorated by co-administration of CBD (Morgan *et al.* 2010*a*; Englund *et al.* 2012). Similarly, skunk was identified as the type of cannabis most strongly associated with paranoia. This is consistent with evidence that the paranoia-inducing effects of THC can also be inhibited by CBD (Englund *et al.* 2012), and that regular skunk use is associated with an increased risk (Di Forti *et al.* 2009) and earlier onset (Di Forti *et al.* 2013) of psychosis, while resin/hashish is not, even in daily users (Di Forti *et al.* 2015).

Although skunk was most clearly defined by these negative effects, it was also rated as having the ‘best high’ and was considered the ‘preferred type’. Clinical and public health interventions related to high-potency cannabis, focusing in its negative effects, should be interpreted in the context of users’ own preferences. Given that skunk was rated as the most available type of cannabis, consistent with previous findings (Hardwick & King, 2008; Potter *et al.* 2008; Freeman *et al.* 2014) people who do prefer it will probably find it easy to obtain in the illicit market. On the other hand, those who do not prefer skunk – or find that its negative effects outweigh the desirable ones – may have little choice due to the current lack of available alternatives. Perhaps varieties of cannabis with weaker effects on memory and paranoia (e.g. other grass, resin) may be more desirable in this respect.

When comparing these lower potency varieties, it is somewhat surprising that resin was rated as having a better high and stronger effects on memory and paranoia, given that it generally contains lower THC and higher CBD than imported herbal cannabis (Hardwick & King, 2008). It may be relevant that variation in cannabinoid content is comparatively greater in resin (Potter *et al.* 2008) and some resin can be highly potent (e.g. 39.3% THC in the Netherlands; Pijlman *et al.* 2005). Such preparations are incredibly rare in the UK (Potter *et al.* 2008) but perhaps experience with especially potent forms of resin could have led some people to rate it as having the best high, and strongest effects on memory and paranoia.

This study has some limitations. First, it used a self-selecting (drug using) sample. This enabled a large number of cannabis users to be recruited, but it does limit the extent to which the findings can be attributed to the general population, or more problematic users, as dependence scores were modest on average. Second, because dependence was estimated using the SDS rather than a structured clinical interview, it was not possible to tease apart specific aspects of cannabis use disorder such as tolerance, withdrawal, craving, failing obligations, giving up other recreational interests, and persistent use in spite of problems. Additionally, the study was cross-sectional and causality cannot be established on the basis of these results. Indeed, it is quite plausible that reverse causation might explain our findings (e.g. as a result of dependence, people use more skunk). Third, skunk was used for more days per month than the other types, which might explain the reported association with dependence. However, variance in days per month of use was similar for each of the three types, suggesting that these data were equally appropriate to detect the existence of possible associations with dependence. Fourth, although we quantified use of three different cannabis preparations, we cannot be sure that the terms we used (e.g. skunk) were meaningful to the population tested (Potter & Chatwin, 2012), although similarly named types were predictive of actual THC and CBD concentrations elsewhere (Freeman *et al.* 2014).

## Conclusion

Use of high-potency (skunk) cannabis is associated with an increased severity of dependence, especially in young people. Skunk is also rated as having stronger effects on memory impairment and paranoia than other types of cannabis, but at the same time it produces the best high and is users’ preferred type.

## Footnotes

† One-eighth of an ounce (3.5 g), an amount typically sold in the UK.
